# An integrated comparative physiology and molecular approach pinpoints mediators of breath-hold capacity in dolphins

**DOI:** 10.1093/emph/eoab036

**Published:** 2021-10-28

**Authors:** Ashley M Blawas, Kathryn E Ware, Emma Schmaltz, Larry Zheng, Jacob Spruance, Austin S Allen, Nicole West, Nicolas Devos, David L Corcoran, Douglas P Nowacek, William C Eward, Andreas Fahlman, Jason A Somarelli

**Affiliations:** 1 Nicholas School of the Environment, Duke University Marine Laboratory, Beaufort, NC, USA; 2 Department of Medicine, Duke University Medical Center, Durham, NC, USA; 3 Dolphin Quest, Oahu, Honolulu, HI, USA; 4 Duke Center for Genomic and Computational Biology, Duke University, Durham, NC, USA; 5 Pratt School of Engineering, Duke University, Durham, NC, USA; 6 Department of Orthopaedic Surgery, Duke University Medical Center, Durham, NC, USA; 7 Duke University Medical Center, Duke Cancer Institute, Durham, NC, USA; 8 Global Diving Research, Inc., Ottawa, ON, Canada; 9 Research Department, Fundación Oceanogrāfic de la Comunitat Valenciana, Valencia, Spain

**Keywords:** ischemic stress tolerance, cetaceans, diving physiology, oceans and human health, ALOX5, lipoxygenase

## Abstract

**Background and objectives:**

Ischemic events, such as ischemic heart disease and stroke, are the number one cause of death globally. Ischemia prevents blood, carrying essential nutrients and oxygen, from reaching tissues, leading to cell and tissue death, and eventual organ failure. While humans are relatively intolerant to ischemic events, other species, such as marine mammals, have evolved a unique tolerance to chronic ischemia/reperfusion during apneic diving. To identify possible molecular features of an increased tolerance for apnea, we examined changes in gene expression in breath-holding dolphins.

**Methodology:**

Here, we capitalized on the adaptations possesed by bottlenose dolphins (*Tursiops truncatus*) for diving as a comparative model of ischemic stress and hypoxia tolerance to identify molecular features associated with breath holding. Given that signals in the blood may influence physiological changes during diving, we used RNA-Seq and enzyme assays to examine time-dependent changes in gene expression in the blood of breath-holding dolphins.

**Results:**

We observed time-dependent upregulation of the arachidonate 5-lipoxygenase (ALOX5) gene and increased lipoxygenase activity during breath holding. ALOX5 has been shown to be activated during hypoxia in rodent models, and its metabolites, leukotrienes, induce vasoconstriction.

**Conclusions and implications:**

The upregulation of ALOX5 mRNA occurred within the calculated aerobic dive limit of the species, suggesting that ALOX5 may play a role in the dolphin’s physiological response to diving, particularly in a pro-inflammatory response to ischemia and in promoting vasoconstriction. These observations pinpoint a potential molecular mechanism by which dolphins, and perhaps other marine mammals, respond to the prolonged breath holds associated with diving.

## Lay Summary

Reductions in blood flow are associated with tissue damage in humans; however, marine mammals have evolved remarkable tolerance to reductions in tissue blood flow during diving. We found that changes in gene expression in breath-holding dolphins may support a response to diving highlighting a potential molecular underpinning for apnea tolerance.

## INTRODUCTION

### Ischemic stress and hypoxia are associated with negative clinical outcomes in humans

Maintenance of homeostatic function in mammalian tissues is directly dependent on a continuous supply of oxygenated blood. Interruption of this blood supply, known as ischemia, results in reduction in local oxygenation compared to normal physiologic levels, or hypoxia, and can lead to inflammation and cell/tissue death in humans [1–5]. In the case of a stroke, disruption of cerebral blood flow can result in cell death at the core of infarction within minutes [6]. Ischemia is the causative factor in multiple clinical settings, and ischemic heart disease is the number one cause of death globally, accounting for over 9 million deaths each year [7, 8].

### Marine mammals experience regular ischemic events

While humans have little tolerance for ischemic stress and hypoxia, a number of species have evolved unique physiologies that allow them to seemingly thrive despite regular tissue-level ischemia and low-oxygen environments. Marine mammals are one group of animals that undergo repeated daily ischemic events. During a dive, a marine mammal experiences a suite of cardiovascular changes that aid in reducing whole-body oxygen (O_2_) demand [9, 10]. As part of this response, both heart rate (*f*_H_) and stroke volume decrease, resulting in reduced cardiac output [11, 12]. Increased peripheral resistance, through selective vasoconstriction, helps assure that mean arterial blood pressure is maintained, at least in studies on forced diving in seals [13, 14]. Ultimately, this response conserves oxygen in the blood and lungs for O_2_-sensitive tissues like the brain and the heart, while the skeletal muscles rely on endogenous myoglobin-bound O_2_ for aerobic metabolism [15, 16]. As the dive continues, however, O_2_ stores are consumed. The hypoxemia that develops during a dive can be extreme—blood O_2_ concentrations as low as 2.7 ml O_2_/dl have been measured in the diving elephant seal. In comparison, the lowest blood O_2_ concentration ever measured in a human of 9 ml O_2_/dl was in a climber near the top of Mount Everest [17, 18]. *In vitro* studies of the seal brain indicate an increased reliance of cerebral tissue on anaerobic metabolism during hypoxia [19]. Similarly, seal neurons demonstrate an ability to continue to discharge four times longer under severely hypoxic conditions compared to mouse neurons [20]. While the responses to submersion in water are largely conserved across all vertebrates, it is clear that many of the physiological adaptations that support diving are exaggerated in marine mammals compared to other taxa to match the demands of extreme hypoxia [21, 22]. These physiological differences highlight the tremendous potential to study marine mammals as model organisms for the investigation of adaptations to ischemic and hypoxic stress tolerance, and the cardiorespiratory plasticity that helps prevent hypertension [11, 12, 23].

### Marine mammals have evolved molecular adaptations to ischemic stress tolerance

Increasing attention has been paid to the defenses marine mammals possess against the oxidant by-products and inflammation associated with ischemic, hypoxia, and reperfusion at the molecular level [24–26]. Elevated levels of heme degradation and concentrations of endogenous carbon monoxide (CO) in northern elephant seals have been suggested to potentially protect against damage from ischemia/reperfusion injury due to the known role of CO in supporting vasodilation and decreasing hypertension [27]. Several studies have highlighted the importance of highly-adapted antioxidant systems in marine mammals for reducing oxidative stress [28, 29] resulting from ischemia/reperfusion secondary to elevated glutathione [30–32], superoxide dismutase [32, 33] and catalase [32]. Using phylogenetic and evolutionary convergence approaches, several gene families have been identified that may contribute to the increased ischemic stress tolerance of marine mammals, including hypoxia-inducible factor 1 (HIF-1) [34–36], genes relating to the glutathione system and peroxiredoxins [27, 37–39] and several genes linked to O_2_ storage, particularly hemoglobin and myoglobin [40–43]. Yet, few studies have examined differential gene expression in marine mammals under conditions of ischemia and hypoxia (i.e. diving conditions).

Here, we investigate the dynamic molecular changes that occur during an apnea in bottlenose dolphins using molecular analysis of peripheral blood mononuclear cells (PBMCs) and serum sampled at regular intervals during breath holds. Dolphins are a particularly tractable and well-studied model for understanding the molecular drivers of diving adaptations. Our integrated analyses pinpoint a gene regulatory network centered around the arachidonate 5-lipoxygenase (ALOX5) gene and its downstream metabolites, leukotrienes, as differentially activated during breath holding. This activation of ALOX5 is consistent with cardiovascular control through a reduction in *f*_H_ and peripheral vasoconstriction to efficiently manage O_2_ use during diving. Based on our collective results, we propose a model in which the ALOX5 pathway is upregulated by blood cells in response to extended breath holds as a mechanism to sustain vasoconstriction and maintain O_2_ stores for critical organs while diving.

## RESULTS

### Analysis of baseline RNA-Seq data from dolphins pinpoints enriched gene regulatory networks

**Figure 1.**
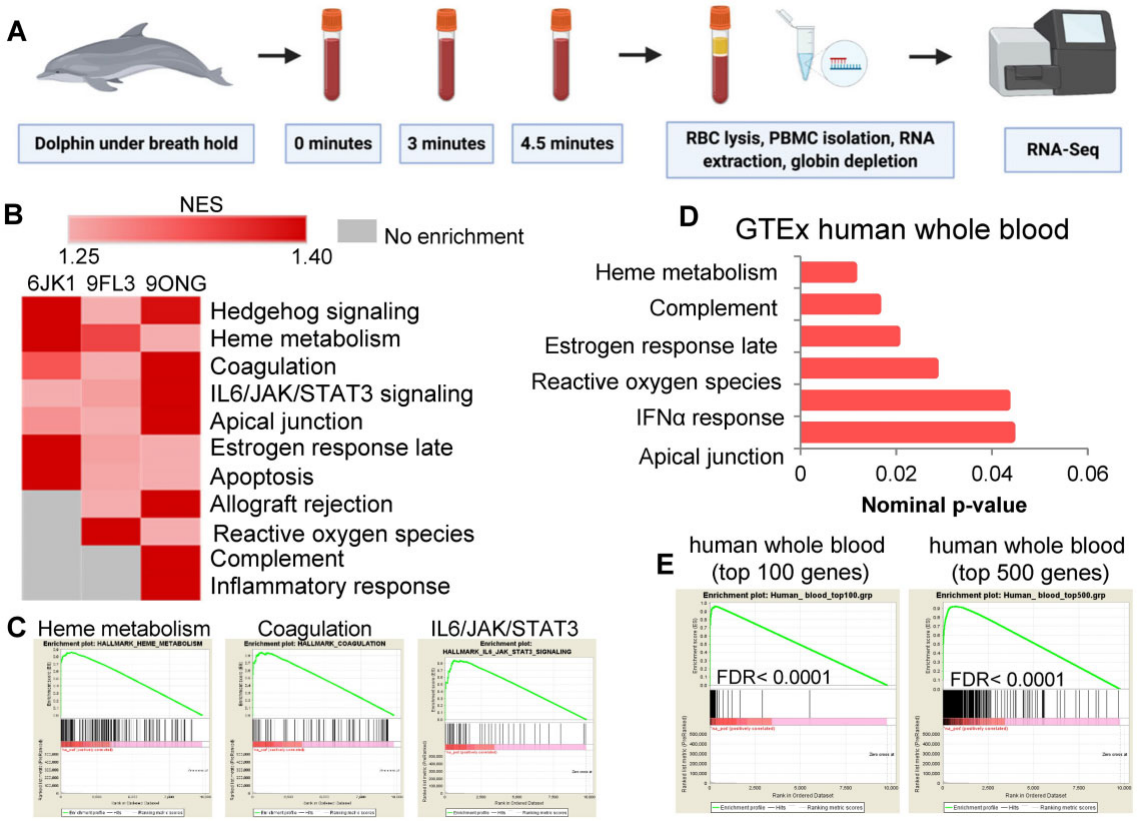
RNA-Seq from dolphin peripheral blood mononuclear cells reveals enrichment of pathways similar to humans. (**A**) Whole blood from dolphins undergoing fasted breath holds at baseline (0–30 s), 3 min, and 4.5 min was collected from tail flukes and stored in PAXgene tubes for RNA extraction of peripheral blood mononuclear cells and RNA-Seq. (**B**) Gene set enrichment analysis of baseline RNA-Seq data ranked by total expression pinpoints highly expressed relevant pathways. (**C**) Enrichment plots for heme metabolism, coagulation and IL6/JAK/STAT3 signaling from baseline dolphin RNA-Seq data. (**D**) GSEA-based pathway enrichment from GTEx human whole blood RNA-Seq data ranked by total expression. (**E**) GSEA enrichment plots comparing dolphin RNA-Seq data ranked by total expression with top 100 and top 500 expressed genes in human whole blood.

All samples produced between 30 and 40 million reads, with no time-dependent changes in read counts across samples (Supplementary Fig. S1A). Principal component analysis and hierarchical clustering of all samples (three individual dolphins × three time points) revealed both individual- and within-individual time-dependent grouping of the data (Supplementary Fig. S1B and C). Analysis of baseline RNA-Seq data by GSEA identified multiple pathways enriched in dolphin PBMCs when ranked by total expression, including hedgehog signaling and several pathways relevant to blood cell metabolism, including heme metabolism, coagulation, IL6/JAK/STAT3 activation, apical junctions, and allograft rejection (Supplementary Fig. 1B and C). GSEA also identified enrichment of pathways related to apical junctions, interferon-alpha response, estrogen response, complement activity and heme metabolism in RNA-Seq data from GTEx human whole blood transcriptomes (Fig. 1D). Comparison of dolphin baseline RNA-Seq data ranked by total expression with the top 100 and 500 most highly expressed genes in human whole blood showed significant enrichment (FDR < 0.0001; Fig. 1E). Together these analyses suggest that significant overlap exists in mRNA expression at both the gene-level and pathway-level between dolphin and human blood at baseline.

### Breath holding induces upregulation of multiple regulatory pathways

**Figure 2.**
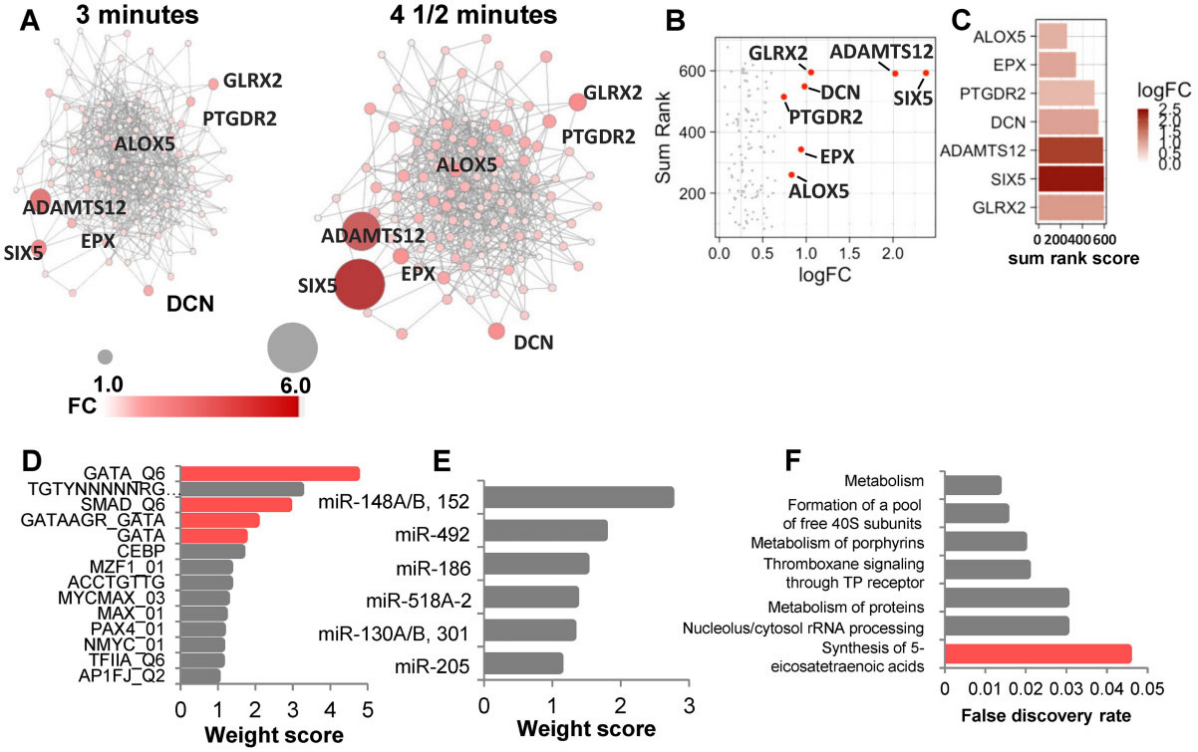
Time-dependent upregulation of gene regulatory pathways during dolphin breath holding. (**A**) Gene regulatory network formed by the time-dependent increases in mRNAs from baseline to 3 and 4.5 min. Fold changes for each gene over time are indicated by darker red and larger nodes. (**B**) Network analysis of genes within a co-expression network with increased expression over time. (**C**) Top genes with increased expression are sorted by their network analysis parameters. (**D**) GeneMANIA-based transcription factor inference pinpoints GATA and SMAD transcription factor targets within the time-dependent network. (**E**) MicroRNA enrichment inference based on the time-dependent network. (**F**) Functional pathway enrichments for the time-dependent gene regulatory network.

We next reasoned that patterns of step-wise increases in mRNA expression may pinpoint molecular responses to breath holding common across individuals. We constructed gene regulatory networks for 136 genes with step-wise increases in mRNA expression from baseline to 3 min and again from 3 min to 4.5 min (Fig. 2A). We performed network analysis to identify genes that are upregulated and have the most network interactions. To do this, we analyzed the time-dependent gene regulatory network for the following parameters: degree, clustering coefficient, closeness, betweenness, neighborhood connectivity and stress. We then plotted the sum rank score of these network parameters with gene expression for each gene in the network. These analyses pinpointed arachidonate 5-lipoxygenase (ALOX5) as among the most connected genes with a time-dependent increase in expression (Fig. 2B and C). Additional genes, including EPX, PTGDR2, SIX5, DCN, ADAMTS12, and GLRX2 demonstrated upregulation and/or high network connectivity (Fig. 2B and C). We used GeneMANIA to infer transcription factor and microRNA targets from this time-dependent network. The gene regulatory network produced from these genes displayed enrichment in targets from several transcription factor families, including GATA and the small, mothers against decapentaplegic (SMAD) families (Fig. 2D), both of which have been implicated in hematopoietic development and regulation [44, 45]. Network inference also pinpointed enrichment of targets of multiple microRNAs, including the miR148A/B/152 family, miR492, miR186, miR518A-2, the miR130A/B/301 family, and miR205 (Fig. 2E). Consistent with the identification of ALOX5 as a core network node, the network was functionally enriched in the synthesis of 5-eicosatetraenoic acid pathway, which is an initial step in the production of arachidonic acid by ALOX5 (Fig. 2F).

### Arachidonate 5-Lipoxygenase (ALOX5) and subsequent lipoxygenase activity is enhanced in breath-holding dolphins

**Figure 3.**
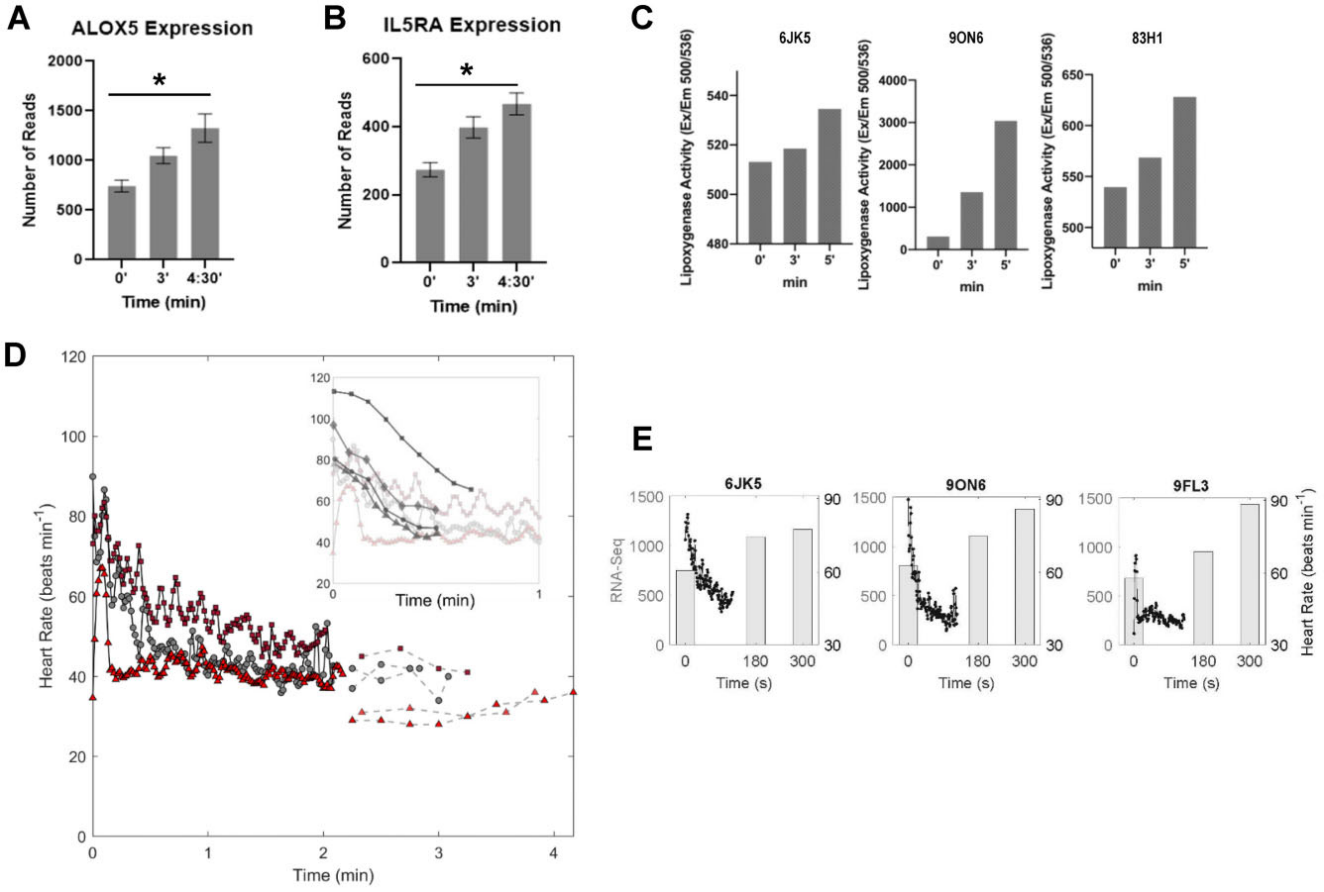
Dolphins induce ALOX5 activity during breath holding. (**A**) ALOX5 and (**B**) IL5RA mRNA expression is significantly increased over time during breath holding. (**C**) Individual dolphin lipoxygenase activity in whole blood was collected at an independent sampling date. (**D**) Physiological measurements of heart rate for three individual dolphins (black lines from ECG data previously published in Blawas *et al*. [23], and dashed lines from echocardiogram data previously published in Fahlman et al., 2020) over time. Inset shows heart rate for humans performing breath holds with facial immersion in water (dark gray in inset) overlaid on dolphin heart rate. Human heart rate traces were digitally extracted from [46–48]. (**E**) Overlay of heart rate data with ALOX activity in three individual dolphins.

At the gene level, ALOX5 was one of just two genes, along with IL5RA, that was significantly upregulated in all three individuals during breath holding (Fig. 3A and B). Lipoxygenase assays from serum of three individual dolphins collected in 2019 revealed time-dependent increases in lipoxygenase activity during breath holding in all three individuals, consistent with the RNA-Seq analyses (Fig. 3C). Comparison of the timing of these molecular changes with previously-published *f*_H_ measurements from the same dolphins demonstrated that changes in gene expression and enzymatic activity were coincident with the expected timing of bradycardia based on the heart rate data (Fig. 3D). Overlay of the RNA-Seq data for ALOX5 mRNA expression with the heart rate data shows the upregulation of ALOX5 is concomitant with lower heart rate (Fig. 3E).

## DISCUSSION

Dolphins and other cetaceans have evolved exquisite physiological adaptations to deal with the challenges of a fully aquatic lifestyle including having a hydrodynamic shape to reduce drag [49], counter-current heat exchangers for thermoregulation [50, 51], and cardiorespiratory plasticity for exquisite management of circulation and respiratory gases [11, 12, 23, 52, 53]. The well-known dive response, a suite of adaptations that support reduced aerobic metabolism during diving, involves apnea, bradycardia, and peripheral vasoconstriction that assures maintained mean arterial blood pressure as blood flow to peripheral tissues is reduced and allows regulation of perfusion to conserve O_2_-rich blood for the brain and heart. To maintain a constant mean arterial blood pressure and prevent hypertension, these adaptations must work in concert to ensure efficient autoregulation; however, extended dives also result in frequent events of ischemia and hypoxia [17, 54–58]. Still, knowledge of the molecular adaptations that contribute to the response to diving, enhanced tolerance to hypoxia and ischemic stress, and prevent reperfusion injury during and following a dive, is rudimentary at best. To address this lack of understanding, we combined analysis of breath-hold responses at the molecular level with existing physiological measurements to define the molecular responses to breath holding in dolphins.

While this study is limited by a small sample size and relatively short breath-hold durations, our analyses provide a proof-of-principle design to study molecular alterations in breath-holding dolphins. Analyses of baseline blood RNA-Seq profiles demonstrates similarity to transcriptional profiles of humans, indicating conserved transcriptional profiles across species. We also identified candidate genes and pathways with time-dependent changes in expression throughout the breath holds that were validated in functional studies using independently collected samples and assays. These molecular changes occurred within the calculated aerobic dive limit (cADL) of bottlenose dolphins—the duration of a dive that can be sustained without requiring anaerobic respiration at the cellular level, which has been estimated to be approximately 6.5 min [59]. It is also worth considering the possibility that changes in gene expression could occur to support specific physiological responses to diving during a dive, and that this gene expression differs when the animal is at the surface. Future studies will be focused on using novel technologies, such as GRO-Seq [60] and others to measure nascent mRNAs, as well as measuring later time points to understand the changes that occur upon recovery from breath holds.

To provide physiological context for these molecular alterations on the time scales observed, we compared molecular changes to changes in previously published *f*_H_ patterns in the same individual dolphins during submerged breath holds [11, 23]. If we assume that the appearance of vasoconstriction is coincident with bradycardia, our data provide evidence of an increase in the expression of a gene in blood cells, ALOX5, known to promote vasoconstriction coincident with the onset of vasoconstriction [61]. As potential first responders to hypoxemia, blood cells may produce signals for extended vasoconstriction to slow O_2_ consumption. Indeed, vasoconstriction, or a narrowing of the blood vessels, has been observed during forced dives and suggested as a mechanism by which marine mammals optimize the use of onboard oxygen stores in the blood and muscle [14, 15, 62]. Given the long assumed link between vasoconstriction and bradycardia in marine mammals, the rapid bradycardia we observed suggests that vasoconstriction was occurring in the dolphins in this study during breath holds [63, 64]. We found that changes in gene expression occurred in all animals during the 5-min breath-hold trials and that the same gene families that were upregulated in the dolphins during breath holds help manage vasoconstriction in mice [65] and humans [66]. It is important to note, however, that these pathways are upregulated during an extended breath hold when the animals are reaching their maximum breath-hold tolerance. We have no evidence that these pathways are activated during single-breath dives in nature, as many of these dives are 30 s–1 min.

Our integrated approach reveals possible molecular underpinnings that may support and act synergistically with the cardiac response to breath holding in bottlenose dolphins. Specifically, we identified candidate genes that may provide defense against ischemic and hypoxic stress in dolphins, including the GATA and SMAD transcription factors, several microRNAs, a disintegrin and metalloproteinase with thrombospondin motifs 12 (ADAMTS12), mitochondrial glutaredoxin-2 (Glrx2) and ALOX5. Interestingly, many of these factors play known roles in regulating hypoxia, hematopoiesis, and ischemic stress responses. For example, the GATA transcription factor family is an important modulator of hematopoietic development of T lymphocytes, mast cells, and erythrocytes [45]. Likewise, the SMAD family regulates hematopoietic stem cells [44]. Of the microRNAs identified from our analysis of target enrichments, nearly all have been shown to be protective against ischemia-induced cell death, including miR148A [67], miR492 [68], miR186 [69–71], miR130 [72], and miR205 [73]. At the protein-coding gene level, ADAMTS12 genetic variation is associated with pediatric stroke [74], GLRX2 is implicated in neuroprotection during hypoxia and ischemia [75], and ALOX5 is known to be induced by hypoxia [76] and mediates the production of pro-inflammatory leukotrienes, which induce bronchoconstriction and vasoconstriction [58] as well as the generation of lipid peroxidases secondary to lipoxygenase production [77]. This suggests that ALOX5 may play a role in an inflammatory cascade in response to changes during breath holding. In addition, both ALOX5 and IL5RA have been identified as susceptibility genes associated with asthma and asthmatic inflammation in humans [78, 79], and a monoclonal antibody to the IL5RA ligand, IL5, is FDA-approved for the treatment of severe eosinophilic asthma [80, 81]. Given the intricate connection between molecular control and physiologic function to manage ischemia, hypoxia and inflammatory responses in humans and rodent models [82], it is intriguing to speculate as to how dolphins and other marine mammals may uncouple or leverage these interconnected processes for improved tolerance to ischemic/hypoxic stress without the pathological consequences associated with hyper-stimulation of these processes.

Our results reveal upregulation of ALOX5 mRNAs and increased lipoxygenase activity in bottlenose dolphins during breath holds. The changes in gene expression and enzyme activity were measured in unpaired breath-hold trials collected in separate years.

By examining molecular data through a physiological lens, these data connect the cellular and tissue-level responses of dolphins to apnea to understand whether the bottlenose dolphin may be genetically tuned to dive and withstand the hypoxia and the potential implications of this to translational medicine. Our results uncover potential candidates at the intersection of ischemia, hypoxia, and vasoconstriction that may contribute to the exquisite adaptation of dolphins and other marine mammals to life in the ocean.

## MATERIALS AND METHODS

### Data collection and animal information

Four adult male bottlenose dolphins (*Tursiops truncatus*) housed at Dolphin Quest Oahu (Honolulu, HI, USA) with an average (± S.D.) age of 22.8 ± 9.9 years (range = 11–35 years) and body mass of 198.1 ± 42.9 kg (range = 147.0–251.7 kg, Table 1) participated in this study. All data were collected under voluntary participation and the animals could end a trial at any time. Routine veterinary assessments include venous blood sampling, and the dolphins that participated in this study had previously been desensitized to the blood sampling protocol. The study protocols were accepted by Dolphin Quest and the Animal Care and Welfare Committee at the Oceanogràfic (OCE-17-16, amendments OCE-29-18 and OCE-3-19i).

**Table 1.** Animal ID, age (years), body mass (kg) and included analyses for all dolphins in the study.

Animal ID	Age (years)	Body mass (kg)	RNA-Seq	Lipoxygenase assay
6JK5	24	200.9	x	x
9FL3	35	251.7	x	
9ON6	21	192.8	x	x
83H1	11	147.0		x
Mean±SD	22.8 ± 9.9	198.1 ± 42.9		

### Experimental trials

Whole blood samples were collected at baseline, 3 min and 4.5–5 min of breath holding on fasted dolphins at Dolphin Quest, Oahu, March 2018 and May 2019. All trials were performed in the morning, when the animals were fasted with at least 12 h having passed since the last meal on the previous day to minimize the potential confounding effect of nutritional state. To ensure that the samples were collected during resting behavior each breath hold was proceeded by 2 min of rest or slow swimming at the surface. A trial was initiated when the dolphin rolled into dorsal recumbency with its blowhole submerged and continued for approximately 5 min (Supplementary Movie S1). The breath hold ended when the animal rolled into ventral recumbency and took a breath (Supplementary Movie S1). Prior to this study, the animals had previously participated in breath-hold experiments of durations up to 5 min [11, 51].

### Blood collection and processing for RNA-Seq

Whole blood was collected from tail flukes at baseline (0–30 seconds into the breath hold) and during breath holding for 3 min and 4.5 (2018) or 5 (2019) min while the animals were in dorsal recumbency with their blowholes submerged (Fig. 1A and Supplementary Movie S1). For RNA-Seq blood was collected into PAXgene tubes, and RNA-Seq was performed after shipping, red blood cell lysis, and RNA extraction (Fig. 1A). All samples were shipped the same day via overnight courier to Duke University for downstream processing. For RNA extraction, tubes were equilibrated to room temperature for 2 h to achieve complete lysis of blood cells. Subsequently, tubes were centrifuged at 4000 × *g* for 10 min. Pellets were resuspended in 4 ml of RNase-free water and RNA was extracted according to the PAXgene Blood RNA kit (PreAnalytiX #762164). Prior to library prep, RNA quality was evaluated on a Bioanalyzer 2100 (Agilent). Stranded mRNA-seq libraries were prepared using the Nugen Universal Plus mRNA-seq Library preparation kit with Globin AnyDeplete (Tecan #9147-A01). Libraries were sequenced at 150 bp paired-end on one lane of an Illumina NovaSeq 6000 instrument S-Prime flow cell. Library preparation and sequencing were performed in conjunction with the Duke University Sequencing and Genomic Technologies Shared Resource. Samples collected in 2018 were used to conduct RNA-Seq analysis and samples collected in 2019 were used for the lipoxygenase assays.

### RNA-Seq data analysis

RNA-seq data were processed using the TrimGalore toolkit [83] which employs Cutadapt [84] to trim low-quality bases and Illumina sequencing adapters from the 3′-end of the reads. Only reads that were 20 nt or longer after trimming were kept for further analysis. Reads were mapped to the turTru1v92 version of the dolphin genome and transcriptome [85] using the STAR RNA-seq alignment tool [86]. Reads were kept for subsequent analysis if they mapped to a single genomic location. Gene counts were compiled using the HTSeq tool [87]. Only genes that had at least 10 reads in any given library were used in subsequent analysis. Normalization and differential expression across the time points were carried out using the DESeq2 [88] Bioconductor [89] package with the R statistical programming environment [90]. The false discovery rate was calculated to control for multiple hypothesis testing. To identify relevant molecular features of dolphin breath holding, we first analyzed the RNA-Seq data from all individuals at baseline using gene set enrichment analysis (GSEA) [91, 92]. GSEA is a standard pathway analysis tool that calculates enrichment scores for annotated pathways based on the rank order of genes present in the data for each pathway. Pathways with genes that are more upregulated or downregulated are more likely to be enriched in a data set than pathways whose genes are randomly distributed throughout the data. Pathway enrichment in dolphin PBMCs at baseline, with genes ranked on total expression value, were compared with human whole blood pathway enrichments from the Genotype-Tissue Expression (GTEx) project.

### Construction of gene regulatory networks

Gene expression networks were created using GeneMANIA [93], implemented within the Cytoscape platform [94]. For time-dependent gene network construction, all nodes with 0 or 1 connection were trimmed out of the networks. Two additional non-coding RNA genes were eliminated (RF00016 and RF00026). To quantify network connectivity, all genes in the network were individually ranked by the following network parameters: degree, clustering coefficient, closeness, betweenness, neighborhood connectivity and stress. These rankings were summed to generate a sum rank score for each gene. Pathway enrichments were performed in STRING using the trimmed network of 123 genes. Human whole blood transcriptomics data used for the analyses described in this manuscript were obtained from the Genotype-Tissue Expression (GTEx) Program Portal (https://gtexportal.org/home/, accessed on 20 September 2020).

### Lipoxygenase assays

Briefly, 5 ml of blood was collected directly into BD Vacutainer^®^ SST™ Tubes (SST) using a 21 g, ¾ in. winged infusion set with a BD Vacutainer adapter and holder. Tubes were gently inverted five times to activate clotting reagent and allowed to clot at room temperature for 30 min in an upright position. Tubes were centrifuged at 1500 × *g* for 15 min to separate serum fractions, and serum was transferred to 15 ml conical tubes, frozen on dry ice, and shipped to Duke University for downstream analyses. Sera were stored at −80°C until use. Lipoxygenase activity was quantified from 1 μg of total protein using a Fluorometric Lipoxygenase Activity Assay Kit (BioVision Inc; cat. #K978). All lipoxygenase activity assays were performed in triplicate biological replicates from three individual dolphins. Differences in lipoxygenase activity across each time point were analyzed using analysis of variance with Tukey’s post-hoc adjustment for multiple testing in Graphpad Prism 8.

## Supplementary data

Supplementary data is available at *EMPH* online.

Conflict of interest: The authors declare no competing or financial interests.

## Supplementary Material

eoab036_Supplementary_Data
