# Supplementary figures and images for: An integrated comparative physiology and molecular approach pinpoints mediators of breath-hold capacity in dolphins
Source: Evol Med Public Health. 2021 Oct 28;9(1):420–30. doi: 10.1093/emph/eoab036 (PMC8833867; doi:10.1093/emph/eoab036)

**A****Read Counts for Individuals at Different Time Points**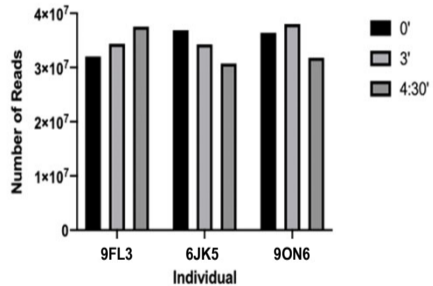**B**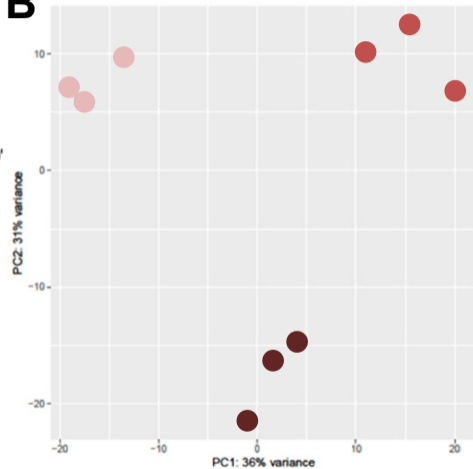**C**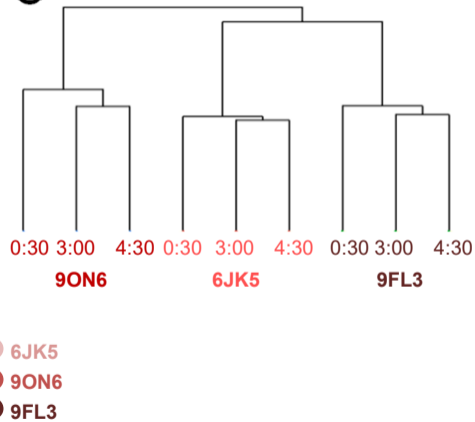

Supplement: eoab036_Supplementary_Data [file eoab036_Supplementary_Data.zip › S1_Fig.pdf]
